# Integrating Cognitive Behavioral Therapy in Dermatology: A Call to Action for Self‐Guided Modules

**DOI:** 10.1111/srt.70095

**Published:** 2024-10-07

**Authors:** Isabella J. Tan, Aarushi K. Parikh, Mohammad Jafferany

**Affiliations:** ^1^ Rutgers Robert Wood Johnson Medical School The State University of New Jersey Piscataway New Jersey USA; ^2^ Department of Psychiatry and Behavioral Sciences Central Michigan University College of Medicine Mount Pleasant Michigan USA

## 1 Background and Scientific Rationale

### 1.1 Cognitive Behavioral Therapy in Dermatology

Cognitive behavioral therapy (CBT) serves as an adjunctive treatment in dermatology to address the psychological impacts of dermatologic conditions [1]. CBT focuses on developing coping strategies, managing stress, and modifying negative thought patterns, thereby improving body image and providing emotional support. Research indicates that incorporating CBT into dermatologic care may enhance psychological outcomes, increase treatment adherence, and improve quality of life [1].

### 1.2 Psychological and Quality of Life Impacts in Dermatologic Conditions Among Skin of Color

Dermatologic conditions such as hidradenitis suppurativa (HS), vitiligo, keloids, and melasma not only manifest physically but also significantly affect psychological well‐being, especially in individuals with skin of color (SOC). Patients with SOC often encounter unique challenges associated with these conditions, including heightened visibility of skin lesions, cultural stigmas surrounding skin appearance, and perceptions of beauty norms that may not align with their ethnic or cultural background. These factors can exacerbate psychological distress, leading to anxiety, depression, and diminished self‐esteem.

### 1.3 Need for Tailored CBT Approaches

Addressing the psychological impacts of dermatologic conditions is crucial alongside treating physical symptoms. CBT, a structured psychotherapeutic approach, offers promising avenues for managing these conditions by helping patients develop coping strategies, manage stressors, and improve overall emotional well‐being [2].

CBT interventions in dermatology focus on disconnecting the emotional responses, such as mood dysregulation and self‐worth, from the visible cosmetic manifestations and physical discomfort which often results from these conditions [2]. By targeting cognitive restructuring and promoting behavioral changes, CBT empowers patients to better manage the challenges associated with their dermatologic conditions.

### 1.4 Growing Importance of Self‐Guided CBT

CBT is becoming increasingly recognized for its structured approach in addressing the psychological impacts of conditions such as keloids, melasma, HS, and vitiligo among individuals having SOC. CBT typically encompasses cognitive restructuring, behavioral activation, and the formulation of individualized coping strategies. It is commonly administered through individual or group sessions, and emerging models incorporate self‐guided modules accessed through online platforms or written materials. Self‐guided CBT often spans approximately 2 weeks of 1‐h self‐paced sessions, providing patients with structured exercises and techniques to practice independently. In dermatology, some self‐guided CBT programs also incorporate brief weekly check‐ins with a psychologist or therapist to monitor progress and provide guidance [1]. These check‐ins, lasting about 15 min, can help reinforce therapeutic goals and address any emerging concerns.

Self‐guided CBT can serve as a viable option in dermatology, offering flexibility and accessibility for patients to engage in therapeutic activities at their own pace and in the privacy of their homes. This approach not only reduces barriers to seeking mental health support but also promotes patient empowerment and autonomy in managing their psychological well‐being alongside their dermatologic therapies.

## 2 Psychosocial Challenges in Dermatologic Conditions in Skin of Color

### 2.1 Hidradenitis Suppurativa

Individuals with HS, a chronic dermatologic condition characterized by recurrent, painful nodules and abscesses, are commonly afflicted by depression, anxiety, insomnia, and self‐disgust due to the persistent and painful nature of the disease [3]. The unpredictable and recurrent flare‐ups exacerbate anxiety and depressive symptoms, leading to a continuous state of stress. The visible manifestations of HS often result in heightened self‐consciousness and social withdrawal (Table 1). Additionally, patients with HS have been shown to exhibit a higher prevalence of substance use disorders, including increased consumption of alcohol, cannabis, and tobacco [4].

**TABLE 1:** Psychological impacts and CBT considerations in dermatologic conditions among skin of color.

Condition	Psychological impact	CBT considerations
Hidradenitis suppurativa	Depression, anxiety, insomnia, and self‐disgust due to chronic pain [3]. Stress from recurrent flare‐ups, social withdrawal, and substance use [4].	Address chronic pain and stress management. Enhance body image and reduce self‐consciousness.
Vitiligo	Distress from progressive skin pigmentation loss leading to disfigurement. Risk of depression recurrence and self‐esteem issues.	Focus on coping with visible changes [5]. Maintain self‐esteem and mood post‐therapy [6].
Keloids	Psychological distress from raised, fibrous scars that extend beyond the original wound site.	Alleviate distress by focusing on the emotional impact of scarring [7]. Combine physical and psychological treatment [7].
Melasma	Stress from hyperpigmented patches potentially leading to depression. Impact on mental health due to visible skin changes.	Manage stress and its potential effect on melanin production [8]. Promote positive self‐image and address psychosocial burden [8].

### 2.2 Vitiligo

Vitiligo is marked by progressive skin depigmentation, which may lead to considerable disfigurement. CBT may aid these patients in managing their condition, potentially positively affecting disease progression and reducing the recurrence of depression [5]. Additionally, CBT has been shown to enhance mood and self‐esteem following therapy [6].

### 2.3 Keloids

Keloids are hypertrophic scars resulting from excessive collagen synthesis during the healing process, leading to thickened, fibrous tissue that extends beyond the original wound margins. Patients with keloids often experience substantial physical and psychological distress. Targeted psychological interventions, employing CBT approaches, have been shown to provide symptomatic relief and mitigate psychosocial impact [7].

### 2.4 Melasma

Persistent stress associated with melasma, characterized by hyperpigmented patches, may contribute to the development of depressive disorders [8]. On a biochemical level, melasma has been shown to influence melanin production through stress‐related pathways involving the hypothalamo‐pituitary axis, which regulates stress responses and has been implicated in depression [8].

## 3 Importance of Developing Self‐Guided CBT Modules in Dermatology

Self‐guided CBT offers a flexible and accessible therapeutic approach, permitting patients to engage in therapeutic exercises at their own pace and convenience. This method empowers individuals to develop coping strategies, challenge negative thoughts, and improve their overall mental well‐being without the constraints of scheduled therapy sessions. It is particularly advantageous for individuals facing barriers to traditional in‐person therapy, such as financial constraints, limited availability of mental health professionals, or stigma, which is prevalent in many SOC communities and may deter seeking mental health care [9]. Self‐guided CBT provides a private and non‐stigmatizing option for managing the emotional challenges associated with visible skin conditions, allowing individuals to address their mental health needs discreetly and at their own pace.

Determining the optimal frequency and duration of self‐guided CBT sessions is essential for maximizing its efficacy. Infrequent sessions may lack the consistent reinforcement needed to achieve meaningful psychological improvements, while overly frequent sessions could contribute to burnout or decreased engagement, particularly among individuals with concurrent work commitments and socioeconomic constraints. Session frequency should be adjusted based on individual needs, with options including one module per week, two modules per week, and one module every 2 weeks, or reading two messages per day [10]. This flexibility is effective in tailoring therapy to the patient's schedule and preferences [10].

The integration of CBT into dermatologic care is critical for addressing the psychological burden of conditions such as HS, vitiligo, keloids, and melasma, particularly in SOC populations. Standard treatment protocols may consider incorporating self‐guided CBT to comprehensively address both physical and psychological needs, and future research should focus on refining self‐guided CBT interventions, evaluating long‐term outcomes, and fostering interdisciplinary collaboration to optimize patient care and quality of life.

## Conflicts of Interest

The authors declare no conflicts of interest.

## Data Availability

Data has been derived from public sources.
